# Smart-Plexer 2.0: Leveraging New Features of Amplification Curves to Enhance the Selection of Multiplex PCR Assays in Multi-Target Identification

**DOI:** 10.1021/acs.analchem.5c01181

**Published:** 2025-07-02

**Authors:** Ke Xu, Luca Miglietta, Piyanate Kesakomol, Alison Holmes, Pantelis Georgiou, Nicolas Moser, Jesus Rodriguez-Manzano

**Affiliations:** † Department of Infectious Disease, Faculty of Medicine, 4615 Imperial College London, London W12 0NN, U.K.; ‡ Department of Electrical and Electronic Engineering, Faculty of Engineering, 4615 Imperial College London, London W12 0NN, U.K.

## Abstract

Multiplex PCR plays a critical role in diagnostics by enabling the detection of multiple targets in a single reaction. However, its use is often limited by the need for multiple fluorescent channels, which are restricted in standard PCR instrumentation. Amplification curve analysis (ACA) is a data-driven multiplexing (DDM) approach that overcomes this limitation by using real-time PCR data and machine learning to differentiate targets in a single-channel, single-well format, without requiring instrument modifications. As part of this DDM strategy, we previously introduced Smart-Plexer 1.0, a tool that simulates multiplex assays using empirical singleplex data to identify optimal assay combinations in silico, maximizing kinetic feature distances between targets to support ACA-based discrimination. While Smart-Plexer 1.0 performs reliably in controlled reactions and offers a strong framework for the ACA assay design, it relies on a single kinetic feature and a median-based distance metric, which limits its accuracy in reactions with variable target concentrations or efficiencies. Here, we present Smart-Plexer 2.0, a more robust and accurate version designed to improve the performance in amplification reactions affected by such variability. This version introduces three new kinetic features that are stable across different template concentrations and uses clustering-based distance measures to better capture the variability between targets. Compared to its predecessor, Smart-Plexer 2.0 reduces accuracy variance by an order of magnitude and improves ACA classification by 1.5 and 1% in retrospective 3-plex and 7-plex assays, respectively. In a multi-experiment, cross-concentration evaluation of a newly developed 7-plex assay, it achieved 97.6% ACA accuracy, confirming its robustness across complex scenarios. Smart-Plexer 2.0 offers a reliable and scalable way to design high-performance multiplex PCR assays using standard real-time PCR instruments.

## Graphical abstract

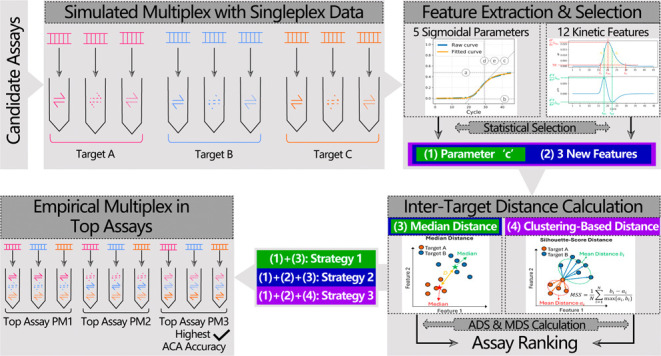

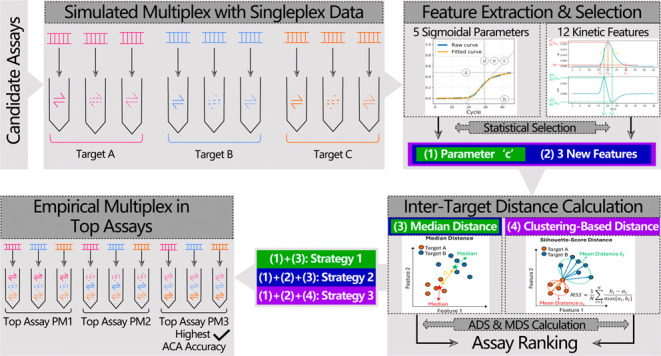

## Introduction

Multiplex polymerase chain reaction (PCR) enables the simultaneous detection of multiple targets in a single reaction, and it is central to molecular diagnostics.^1^ For example, multiplex PCR assays identify and differentiate respiratory viruses with overlapping clinical symptoms, enhancing diagnostic efficiency.^2^ However, conventional multiplexing methods require multichannel fluorescent instrumentation and are constrained by the limited number of available detection channels, restricting scalability.^3^ Single-channel multiplex PCR addresses these challenges by allowing multitarget detection in one reaction, reducing cost and sample volume input. Current methods include: (1) Melting Curve Analysis (MCA), which relies on high-resolution melting peaks to identify targets but is limited by instrument precision, amplification chemistry optimization, and incompatibility with isothermal point-of-care platforms;^4^ (2) Final fluorescent intensity (FFI) modulation, which uses probe concentration tuning to generate amplification curves with separatable plateaus, challenged by low sensitivity and reliability in clinical samples;^5^ (3) Physical Partitioning, which isolates target-specific primers and probes in discrete chambers to prevent cross-reactivity and spectral overlap, but requires custom hardware.^6^ These limitations highlight the need for a robust, cost-effective, and easy-to-use single-well, single-channel multiplex PCR method compatible with existing PCR equipment.^3^

To overcome these limitations, data-driven multiplexing (DDM) enables simultaneous detection of multiple targets with improved specificity and efficiency.^3^ By leveraging data analytics, DDM frameworks improve multiplex PCR assay development by optimizing primer set selection and reducing the cross-reactivity challenges typical in traditional multiplexing. A key innovation within the DDM paradigm is amplification curve analysis (ACA),^7,8^ which leverages unique kinetic signatures of amplification curves (AC) to identify multiple targets in single-well, single-channel reactions.^7,9^ Here, “single-well” defines reactions that mix all of the targets’ primers and probes in a well (e.g., real-time qPCR) or compartment (e.g., digital PCR) and identify targets based on the output of only this well. ACA extracts hand-crafted or network features from amplification curves and employs statistical learning^7^ or deep learning approaches^9,10^ to classify targets, significantly reducing the complexity, cost, and hardware requirements associated with conventional multiplexing.^11^ Its efficacy has been demonstrated across diverse applications, including the detection of respiratory viruses^12^ and carbapenem-resistant genes.^13^

A primary challenge in the ACA is designing optimal assays capable of distinguishing multiple targets based on amplification curve shapes, particularly as the number of targets increases. Identifying optimal assays requires extensive experimental validation, with the testing burden growing exponentially as the number of targets increases.^12^ To address this challenge, our group previously introduced Smart-Plexer, a computational framework designed to streamline multiplex PCR assay development through in-silico optimization of primer mixes.^12^ As shown in Figure 1, with several candidate assays for each DNA target, this framework begins by experimentally applying singleplex assays and leveraging their amplification curves to simulate multiplex reactions, assuming that kinetic information will be maintained when assays are transferred from singleplex to multiplex environments. Numerical kinetic features are then extracted from these ACs and statistically selected, representing the ACs in a reduced dimensional space, and are used to calculate intertarget distances. Primer mixes with larger average (Average Distance Score, ADS) and minimal intertarget distances (minimum distance score, MDS) are ranked higher and considered optimal for ACA performance.^3^ Several high-ranking primer mixes are tested empirically to determine the best one with the highest target classification accuracy. This simulation-driven approach significantly reduces extensive wet-lab testing, accelerating assay development and minimizing costs.

**Figure 1:**
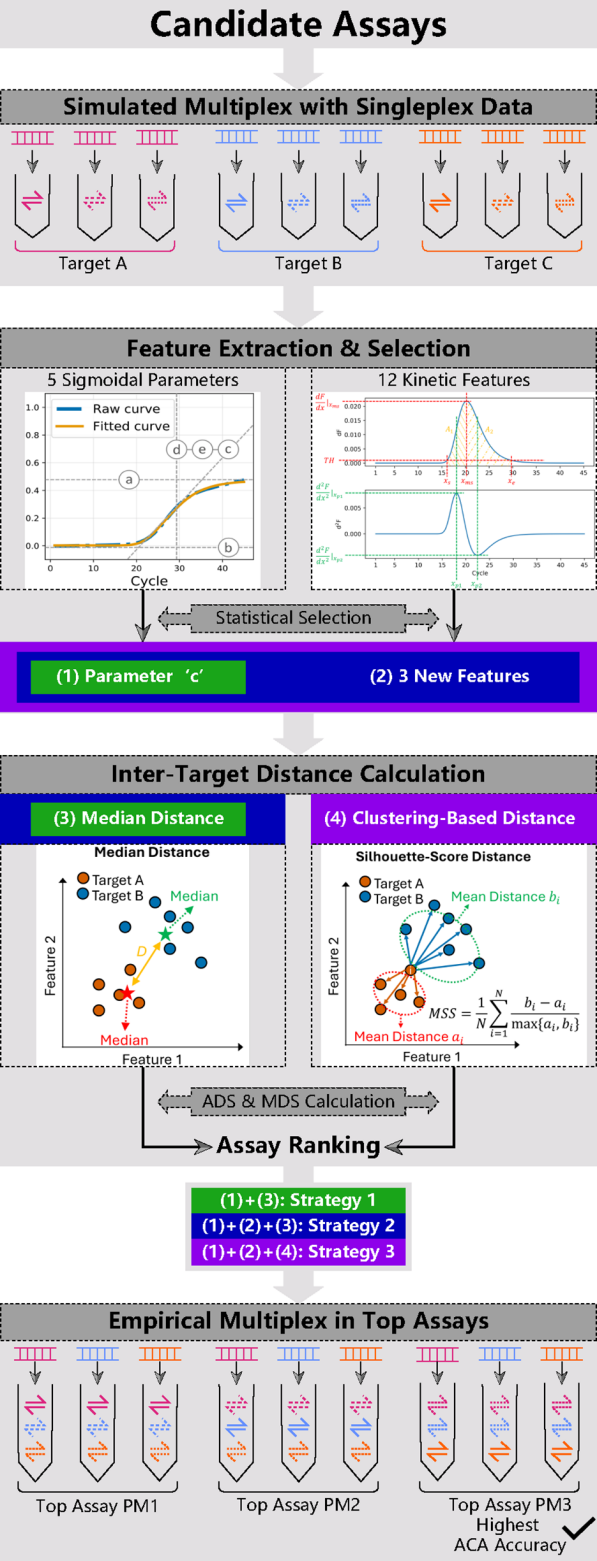
Framework of Smart-Plexer and differences between Smart-Plexer 1.0 and 2.0. Smart-Plexer includes four steps: multiplex simulation, feature extraction & selection, inter-target distance calculation, and empirical multiplex in Top Assays. Smart-Plexer 1.0 uses only (1) “*c”* parameter and calculates (3) median distances among target clusters, ignoring inner-cluster distributions. Smart-Plexer 2.0 leverages (2) three new features along with ‘*c*’ (Strategy 2), and (4) clustering-based distance which measures whole-cluster proximity (Strategy 3).

In our previous research (Smart-Plexer 1.0), a five-parameter asymmetrical sigmoidal model was used to fit the amplification curves, and a single fitting parameter ‘*c*’, which correlates with the curve slope, was selected to calculate intertarget distances, as shown in Figure 1 (1).^12,14^ Although robust and reliable in controlled reactions, this single-feature approach limits performance under complex conditions, such as PCR inhibition, variable primer efficiency, and fluctuating target concentration,^15,16^ in which the changes of reaction efficiency will result in distorted AC shapes. In these situations, plateau shifts and altered slopes of curves may distort ‘*c*’ values, potentially compromising the accuracy of intertarget distance measurement. Furthermore, Smart-Plexer 1.0 utilized median distance, as depicted in Figure 1 (3), which considers only intertarget average distances without addressing inner-target distributions, leading to potential imprecise ADS and MDS. The original reported process combining Steps (1) and (3) is referred to as Strategy 1 (S1).

This study introduces Smart-Plexer 2.0, an enhanced framework designed to address these limitations and improve the assay reliability in challenging scenarios. This new version introduces two key advancements: (1) Enhanced Feature Extraction: We developed 12 novel kinetic features based on biological insights from PCR fluorescent readouts. Statistical evaluations were then performed to select the optimal features that are most distinguishable across targets, as shown in Figure 1 (2). These parameters complement the slope-related ‘*c*’ parameter reported in Smart-Plexer 1,0, significantly enhancing the selection process for multiplex assays by introducing additional kinetic information. (2) Advanced Distance Measurement: We proposed a novel method for distance measurement that considers the intertarget-cluster distances, evaluating relationships between target distributions, as shown in Figure 1 (4). Integrating these innovations, we established two new ACA assay selection strategies: Strategy 2 (S2), which combines the enhanced feature sets with conventional Median Distance calculation, and Strategy 3 (S3), which leverages enhanced features and Clustering-based Distance calculation. Introducing both S2 and S3 allows for the comparison of different distance measuring methods.

Using two independent retrospective data sets, we demonstrated the robustness of the new features against significant target concentration variations, confirming their stability from singleplex to multiplex settings. Comparing old (S1) and new strategies (S2 and S3) in designing a 3-plex assay revealed that S2 and S3 outperform S1 statistically, demonstrating superior performance in selecting optimal assays and avoiding unpromising results with a 10-fold reduced Percentile Interval in accuracy distributions for selected top assays. Furthermore, cross-concentration and multi-experiment tests in a newly developed 7-plex assay indicated that assays selected using S3 showed superior ACA performance, including a 1% improvement in average classification accuracy and an 8.41% narrower accuracy distribution, confirming robustness in complex experimental conditions. This advancement in feature extraction and distance measurement strategies enhances the Smart-Plexer framework, marking a significant step forward in developing reliable and efficient multiplex PCR assays for ACA methodologies and strengthening the capability of high-level multiplexing.

## Experimental Section

This section presents the methods used to develop and validate the enhanced Smart-Plexer strategies. After presenting data used in this study, we introduce the design and selection, and validation of the new features, followed by the description of the new Clustering-based Distance and Smart-Plexer implementation strategies, and then summarize the evaluation carried out for comparison between Smart-Plexer 1.0 and 2.0.

### Data Sets Used in This Study

To ensure robustness, both retrospective and newly generated data sets with cross-platform, cross-experiment, and cross-concentration characteristics were employed. Detailed descriptions can be found in Supporting Information Sections S1, S2 and Table S1.

1. The 7-plex respiratory tract infection (RTI) data set^12^ (SP1-7-plex-RTI): It contains reactions of 7 respiratory pathogens in a single-channel real-time digital PCR (qdPCR), used for developing and selecting optimal features from the amplification curve (AC).
2. External concentration-efficiency data set^17^ (Ex-Conc-Eff): Real-time PCR (qPCR) reactions were conducted with the MT-ND1 gene over a range of input DNA with several master mix quantities, mimicking the amplification efficiency reduction in multiplex reactions. It is used to validate the robustness and consistency of the proposed new kinetic features across concentrations in extreme reaction conditions.
3. The 3-plex RTI data set (SP1-3-plex-RTI): The three targets were from our previous Smart-Plexer research.^12^ This 3-plex RTI data set was used for statistical evaluation of the previous and the new Smart-Plexer strategies over different AC features and distance calculations.
4. New 7-plex RTI data set (SP2-7-plex-RTI): We applied both the original and proposed new strategies on a new multiplex panel of 7 respiratory pathogens. After applying the old and new Smart-Plexer strategies, six optimal multiplex assays were selected, for which single-well multiplex reactions were performed in qdPCR with a range of different target concentrations.

To align with our research interests, we selected different categories of RTI pathogens for this research. Although these respiratory pathogens present with similar early symptoms, they can lead to varying degrees of disease severity and distinct prognoses. Early and accurate identification enables targeted clinical treatment, improving patient outcomes and supporting effective epidemic surveillance, particularly during pandemics.

Our previously proposed adaptive mapping filtering,^13^ an unsupervised preprocessing algorithm for eliminating NTC, flat curves, nonspecific, and low-efficient reactions from qdPCR amplification curves, was applied to all the qdPCR data sets mentioned above.

### New Features from the Amplification Curve

Table S2 lists the 12 new features dedicated to this research, detailing their definitions and mathematical/biological descriptions.

The calculation of these features relies on a 5-parameter sigmoidal fitting of the amplification curve:

$$F(t,p)=\frac{F_{m}}{(1+e^{-S_{c}(t-C_{s})}{)}^{As}}+F_{b}$$ 1

$$p=[F_{m},F_{b},S_{c},C_{s},As{]}^{T}$$ 2

where *t* is the amplification time (or PCR cycle), ***p*** is the parameter vector, *F*(*t*, ***p***) is the fluorescence value, *F*_*m*_ indicates the maximum fluorescence, *F*_*b*_ indicates the baseline fluorescence, *S*_*c*_ is related to the maximum slope of the AC, *C*_*s*_ is linked to the cycle threshold (*C*_t_) value, *As* is the asymmetrical index. The parameter naming was updated to show clear kinetic meanings, and in the new equation naming system, the previous ‘*c*’ parameter is therefore called ‘*S*_*c*_’.

In Smart-Plexer 1.0, the *S*_*c*_ (or ‘*c*’) parameter was initially identified as a robust feature when transferring from singleplex to multiplex reaction conditions, and we leveraged its value for evaluating the intertarget distances in our previous research.^12^ This parameter, though effective, represented a dimensionality reduction (e.g., relying on a single feature), potentially overlooking additional information that could improve multiplex assay optimization. The challenge of extending feature sets lies in balancing the enhancement of multitarget classification with the reliability and repeatability of the features, given the inherent variability of PCR-based biological events.

Therefore, this study further explored 12 new features extracted from the amplification curve and its first and second derivatives. To make the original and the derivative curves smooth enough for reliably extracting kinetic features, the 5-parameter sigmoid fitting was first applied to the raw observation of AC. Nonlinear least-squares optimizations were applied to acquire the optimal estimations of ***p***, which were later used for calculating the first and second derivatives of AC with the following equations:^14^

$$F^{\prime }(t)=\frac{\partial F(t,p)}{\partial t}=\frac{F_{m}S_{c}A_{s}e^{-S_{c}(t-C_{s})}}{(1+e^{-S_{c}(t-C_{s})}{)}^{A_{s}+1}}$$ 3

$$F^{\prime \prime }(t)=\frac{\partial F^{2}(t,p)}{\partial t^{2}}=\frac{F_{m}{S_{c}}^{2}A_{s}e^{-S_{c}(t-C_{s})}(A_{s}e^{-S_{c}(t-C_{s})}-1)}{(1+e^{-S_{c}(t-C_{s})}{)}^{A_{s}+2}}$$ 4

Figure 2a illustrates a raw amplification curve, its fitted curve, and the corresponding first and second derivative shapes, highlighting fiducial points and extracted features. The raw curve is precisely fitted and smoothed, while kinetic information is kept. The maximum peak location and height of *F*′(*t*) are picked as *x*_*ms*_ and $\frac{dy}{dx}|x_{ms}$ . By setting a small threshold *TH* on the first derivative, the *TH*-crossing points *x*_*s*_ and *x*_*e*_ can be identified, representing the beginning of the exponential phase and the beginning of the plateau phase, respectively. Locations of both the positive and negative peaks of the second derivative were also extracted as *x*_*p*1_ and *x*_*p*2_, which are linked to the accelerating and plateauing period of AC.

**Figure 2:**
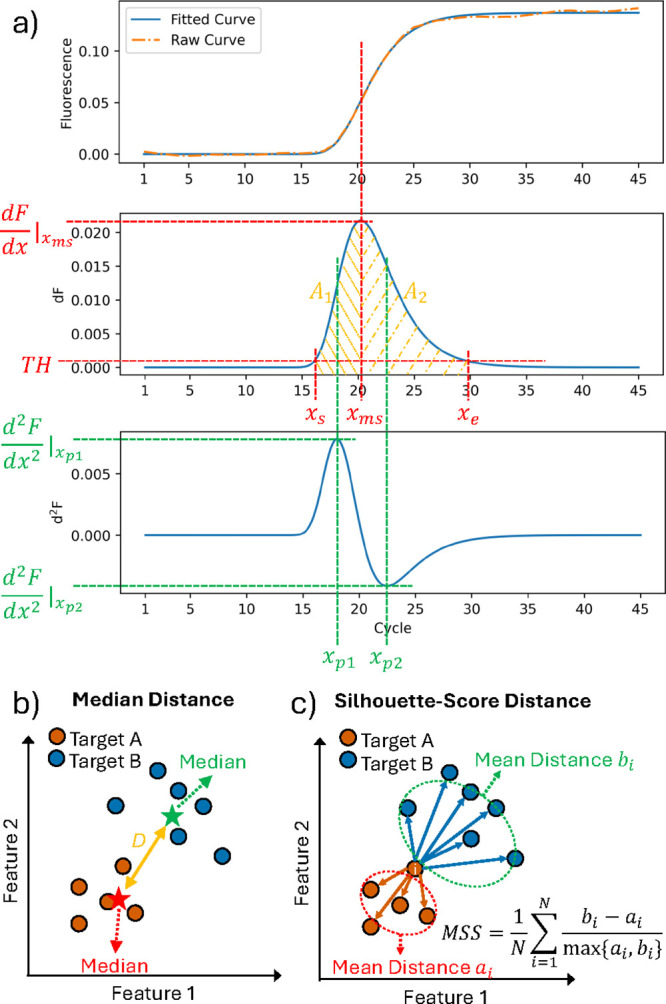
(a) Extraction of fiducial points and kinetic features from the fitted amplification curve (top), first derivative (middle), and second derivative (bottom). (b) Median distance calculation using median points of targets in the feature space. (c) Calculation of clustering-based mean silhouette score (MSS), using both intra- and intertarget Euclidean distances to measure cluster separation between targets A and B.

Provided these fiducial points, 12 new features were designed and extracted, as detailed in Table S2. A group of features focuses on measuring the increasing speed of fluorescence before saturation, e.g., $x_{e}-x_{s},\;x_{p2}-x_{p1},\;\frac{df}{dx}{|}_{x_{ms}},\;\frac{d^{2}f}{dx^{2}}{|}_{x_{p1}},\;\frac{d^{2}f}{dx^{2}}{|}_{x_{p2}}$ , whereas others aim to evaluate the degree of asymmetry between the exponentially increasing phase (period before *x*_*ms*_) and the plateauing phase (period after *x*_*ms*_), e.g., $\frac{x_{e}-x_{ms}}{x_{ms}-x_{s}},\;\frac{A_{2}}{A_{1}},\;-\frac{\frac{d^{2}f}{dx^{2}}{|}_{x_{p1}}}{\frac{d^{2}f}{dx^{2}}{|}_{x_{p2}}}$ .

Instead of looking only at *S*_*c*_ (‘*c*’ parameter in previous publications, which is related to maximum slope), this set of new features describes the AC from multiple perspectives, and takes not only the slope but also the higher-level changing trends and asymmetry into consideration, providing a wider range of kinetic information. These 12 features, together with the five fitted parameters ***p***, pave the way for the following steps of optimizing the Smart-Plexer strategy.

### Feature Selection and Validation

Within the 12 new features plus the five parameters (17 features in total), we need to select and validate those satisfying the following criteria:

**Criterion 1:** Features should be significantly distinguishable across targets; in that case, they could be used to calculate Average Distance Score (ADS) and Minimum Distance Score (MDS), which are vital for the Smart-Plexer workflow.^12^

**Criterion 2:** Features should be robust across concentrations under a wide range of reaction efficiencies; in that case, the unknown sample concentrations will not affect the features’ distributions.

**Criterion 3:** Feature distributions should be kept or at least linearly correlated when the reaction conditions are transferred from singleplex to multiplex. In that case, the ADS and MDS of simulated and empirical multiplexes will remain strongly correlated. Thus, using singleplex-simulated ADS and MDS will not affect the features’ capability of predicting optimal assays, which is vital for Smart-Plexer to work.

To select based on Criterion 1, the 17 features were extracted on the SP1-7-plex-RTI data set, and mean silhouette scores (MSS) were calculated for each feature across all seven targets using the following equation:^18,19^

$$\mathrm{MSS}=\frac{1}{N}\sum_{i=1}^{N}\frac{b_{i}-a_{i}}{\max\{a_{i},b_{i}\}}$$ 5

where *N* is the total number of amplification curves in the data set after AMF, and for each data point *i*, *a*_*i*_ is the mean of the Euclidean Distances between *i* and all other data points within the same target category as *i*, *b*_*i*_ is the mean of the Euclidean Distances between *i* and all the data points within the specific target category which is the closest to *i*. Here, groupings were performed with nature labels of targets when calculating MSS. The closer the inner-target data points and the farther the intertarget data points are, the higher the MSS value will be. Therefore, a feature with a higher MSS value indicates it is easier to distinguish targets using this feature and is optimal for calculating ADS and MDS.

Second, to validate the cross-concentration robustness of the features, analyses were applied to each of the 17 features extracted from the Ex-Conc-Eff data set, where the features’ distribution consistency across seven concentrations under decreasing reaction efficiencies was evaluated using the Kruskal–Wallis test,^20^ which is the nonparametric version of ANOVA. Each concentration group is considered an independent observation of feature values, and the Kruskal–Wallis test was applied on seven groups of observations to detect significant differences in data distributions across groups.

Lastly, we studied the correlation between the features of the singleplex and multiplex reaction conditions. We leveraged the SP2-7-plex-RTI data set since it has both observations from singleplex and multiplex reactions. For each primer set (including the probe) and a given feature, we calculated the feature values from the median curves of both the given primer set’s singleplex and the corresponding multiplex reactions. Please note that in multiplex reactions, the given primer set is mixed with other targets’ primers, resulting in a potentially lower reaction efficiency because of additional thermodynamic interactions.^21,22^ Correlation analyses, including regression plots and Pearson correlation coefficients (*R*), were applied to these paired singleplex and multiplex features.

### Clustering-Based Distance Measurement and New Smart-Plexer Strategies

Using the selected feature vector, we introduced a Clustering-Based Distance Measurement approach to calculate distances between different targets. Previously, as shown in Figure 2b, distances were measured by using only average (median) values, neglecting inner-target distribution variations. This limitation could result in artificially large distance values even if clusters overlap or have significant internal variance, causing imprecise ADS and MDS calculations. To overcome this, we adopted the mean silhouette score (MSS) as the distance measurement among clusters of feature vectors, as illustrated in Figure 2c, considering both inter- and intratarget distributions, thus providing a comprehensive representation of relationships between target clusters and ensuring more precise ADS and MDS calculations. Data points of feature vectors were clustered based on target labels.

We then proposed three strategies for the Smart-Plexer workflow:

1. Strategy 1 (S1): The original Smart-Plexer 1.0, where the feature vector has only one element, the ‘*c’* parameter, and median distances were used for deriving ADS and MDS.^12^
2. Strategy 2 (S2): The feature vector contains all the selected features, and median distances were used for deriving ADS and MDS. Note that we standardized the mean of each feature to 0 and the standard deviation (STD) to 1 before calculating distances.
3. Strategy 3 (S3): The feature vector contains all the selected features, and clustering-based distances were used for deriving ADS and MDS. Note that we standardized the mean of each feature to 0 and the standard deviation (STD) to 1 before calculating distances.

### Evaluation of New Strategies

We compared the performance of S2 and S3 vs S1 using the SP1-3-plex-RTI data set, with which we can test in silico on all the assay combinations. The three strategies were applied to yield optimal primer mixes, respectively, and the Amplification Curve Analysis (ACA) performance in classifying three targets was evaluated using our previously published method,^13^ with 30-fold cross-validation. By analyzing the classification accuracies of each panel (which includes 770 AC in the qdPCR platform), new strategies can be compared to the original one.

Furthermore, S1 and S3 were chosen to help develop a new seven-plex assay (SP2-7-plex-RTI). Using the singleplex data, the two strategies generated their own measurement of ADS and MDS, based on which we selected the top 3 combinations for each strategy and tested empirically in multiplex conditions. ACA using leave-one-concentration-out (LOCO) was then conducted on all of the chosen primer mixes, and statistical analyses were applied to the accuracies of each panel to determine the advantages of S3 over S1. We demonstrated this cross-concentration multi-experiment test to validate the new strategy’s performance under the influence of complex experimental factors.

### Algorithm Implementation

Data analysis was conducted on a MacBook Pro (M1 Pro, 32G RAM, Apple Inc.) with Python 3.9.17. Requirements for key packages of the development environment are listed in Table S3.

## Results and Discussion

### Selected Features and Their Robustness

The feature distributions across concentrations on the SP1-7-plex-RTI data set are presented in Figure S1 and partially depicted in Figure 3a. Most features show large inner-target variances and small intertarget distances, complicating target classification based on them. However, several features, including $\frac{df}{dx}{|}_{x_{ms}},\;\frac{d^{2}f}{dx^{2}}{|}_{x_{p1}},\;\mathrm{and}\;-\frac{d^{2}f}{dx^{2}}{|}_{x_{p2}}$ , exhibited significantly narrower intratarget distributions and clear intertarget distinctions. This intuitive impression was endorsed by their considerably higher Mean Silhouette Scores (MSS), where $\frac{df}{dx}{|}_{x_{ms}},\;\frac{d^{2}f}{dx^{2}}{|}_{x_{p1}},\;-\frac{d^{2}f}{dx^{2}}{|}_{x_{p2}},\;x_{p2}-x_{p1}$ are showing more than twice the MSS values of all other features, along with *S*_*c*_, whose efficacy was discussed in Smart-Plexer 1.0.^12^ $\frac{df}{dx}{|}_{x_{ms}},\;\frac{d^{2}f}{dx^{2}}{|}_{x_{p1}},\;-\frac{d^{2}f}{dx^{2}}{|}_{x_{p2}}$ are new features correlating to the maximal reaction speed and the higher-level trend of increasing and decreasing during the beginning and ending phase of the reaction, describing changes in reaction efficiency and asymmetry specific to each target; thus, they function as indicators of target differences. *x*_*p*2_–*x*_*p*1_ shows good robustness for most targets but is performing unsatisfactorily for HAdV; therefore, it is excluded from further consideration.

**Figure 3:**
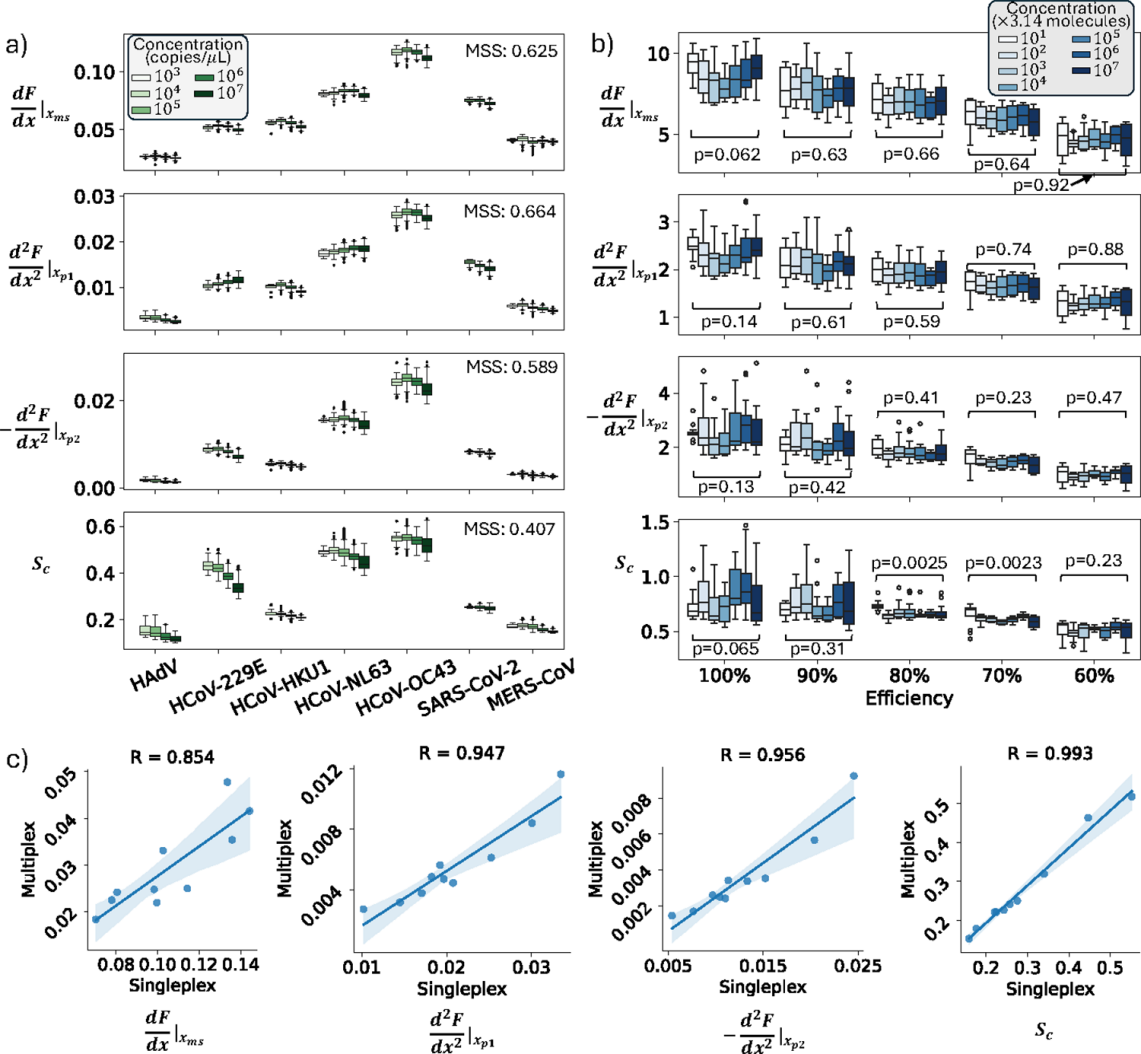
(a) Box plots of four selected features from the SP1-7-plex-RTI data set. *X*-axis shows target categories, with distributions of target concentrations depicted in color shades and grouped in subplots. MSS among target clusters are presented. (b) Box plots illustrating feature distributions across reaction efficiencies (*X*-axis) and concentrations (shaded colors), from the Ex-Conc-Eff data set, with p-values of Kruskal–Wallis tests among concentrations presented. Standard boxplots were used in (a) and (b) where the box spans from Q1 to Q3, whisker extends to the 1.5*IQR from Q1 and Q3, and outliers are depicted in dots. (c) Correlation plots and coefficients between Singleplex and Multiplex-extracted features, where translucent bands indicate a 95% confidence interval.

The features were tested on the Ex-Conc-Eff data set, and their distribution consistency among concentrations under different reaction efficiencies can be seen in Figure S2, a subset of which is depicted in Figure 3b. The three new features demonstrated robustness against concentration variation, as shown by Kruskal–Wallis tests (*p*-values significantly above the 0.01 threshold), confirming that distributions remain stable across varied concentrations. This concentration-robustness is valid across all of the reaction efficiencies, implying that the features may remain concentration-irrelevant when changing the reaction environment from singleplex to multiplex. Although *S*_*c*_ showed slight sensitivity to extremely low concentrations, indicated by lower robustness in extreme conditions, its performance remained stable in typical conditions (posthoc Dunn’s tests confirm the most significant differences only between the lowest concentration group and the rest). This emphasizes the idea of extending feature dimensions, as a single feature can be unreliable under certain conditions, whereas ensemble strategies avoid the solo dependence on potential extreme values. Considering that *S*_*c*_ has good performance in all other concentrations except the lowest and was previously involved in Smart-Plexer, we kept it in the feature vector for the following tests.

Finally, we analyzed the singleplex-multiplex correlations of the selected four features $\frac{df}{dx}{|}_{x_{ms}},\;\frac{d^{2}f}{dx^{2}}{|}_{x_{p1}},\;-\frac{d^{2}f}{dx^{2}}{|}_{x_{p2}}$ and *S*_*c*_, as presented in Figure 3c. All of the features correlate with narrow confidence intervals and R values higher than 0.85. *S*_*c*_ shows the highest R, indicating excellent information consistency between single- and multiplex, which was also validated in our previous paper.^12^ For most features, the absolute values drop when switching from singleplex to multiplex environments. This phenomenon is consistent with the observations in Figure 3b, giving the assumption that in multiplex conditions, the reaction efficiency for each target is decreasing.^21,22^ However, a drop does not affect the simulated ADS and MDS, as features are standardized before calculating distances. Therefore, simulated ADS and MDS always show good consistency with their empirical versions.

### Evaluation of Three Strategies for Smart-Plexer in 3 Plex

Testing results of S1, S2, and S3 on the SP1-3-plex-RTI data set are presented in Figure 4, in which (a–c) show distance maps under S1–S3, plotting locations of primer mixes with simulated ADS and MDS on the x- and *y*-axis, respectively.^12^ Top combinations with both high ADS and MDS have clearly distinguishable AC, clustering toward the map’s top-right corner, while less optimal combinations cluster at the bottom-left. We also plotted the empirical ACA classification accuracy in shades of color on the maps.

**Figure 4:**
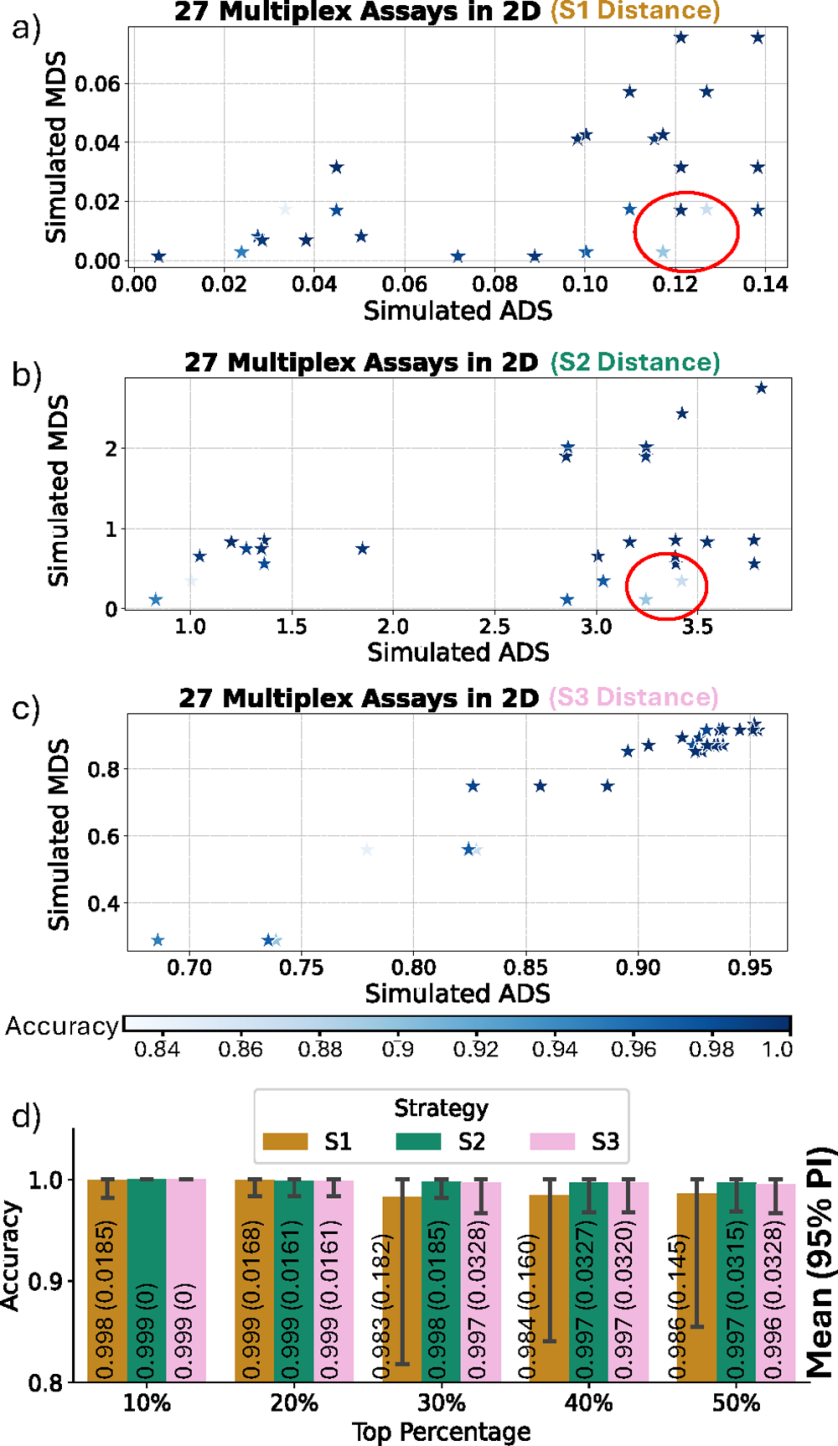
Distance maps under (a) S1, (b) S2, and (c) S3, with ACA accuracies of multiplexes marked with shades of colors. Multiplexes with unexpectedly low ACA accuracies are circled in (a) and (b). (d) Bar plots of ACA accuracies when picking the top 10%–50% assays using the three strategies, with the mean of panel accuracies shown by the bar height and 95% Percentile Interval (PI) demonstrated by the error bar.

Most assays provide high-performance ACA, probably due to the relatively easy task of classifying only three targets. However, certain assays predicted by S1 and S2 as high-performing (right side of maps, Figure 4a,b empirically yielded lower accuracy (<90%), highlighting inconsistencies between ADS-MDS predictions and empirical outcomes. Such inconsistency can result in unexpectedly low accuracy in the selected optimal assays, potentially harming the overall stability of Smart-Plexer. The S3, with the newly proposed MSS distance measurement technique, shows a unique pattern where all of the high-accuracy assays are located in the upper-right corner and are keeping adequate distances from the bottom ones, as depicted in Figure 4c. This sparse distance pattern benefits the top-assay selection by limiting the chance of highly ranking unsatisfactory combinations showing up.

Quantitative performances are shown in Figure 4d, in which we provided the mean and 95% Percentile Interval (PI) of panel accuracies when picking the top 10–50% assays using the three strategies. Although the top 10–20% selections of S2 and S3 do not show significant improvement in mean accuracy compared to S1, they do have smaller PI. When selecting the top 30–50%, S2 and S3 consistently outperformed S1, achieving higher mean accuracies and significantly narrower PIs, confirming enhanced reliability of assay selection with a higher chance of picking optimal assays. The ranking-accuracy mapping presented in Figure S3 also strengthens the assumption that increasing feature dimensions and optimizing distance calculation will ensure a higher chance of picking good combinations and avoiding unpromising ones. In Figure S3, S2 and S3 tend to have low-accuracy assays ranked lower, whereas S1 may accidentally promote low-performance combinations to the top, harming the overall robustness of it.

### Developing a New Multiplex Assay

In the previous section, we concluded that S2 and S3 can provide a more reliable performance in assay selection than S1. We further validated these strategies on a new 7-plex design, generating the SP2-7-plex-RTI data set. The 4 top primer mixes (PM7.13, PM7.14, PM7.77, PM7.78) of S1 and the 3 top primer mixes (PM7.14, PM7.16, PM7.80) of S3 were selected, respectively, based on the singleplex data. Although we aimed to select the top 3 for each strategy, two primer mixes were ranked in third place simultaneously by S1, which is possible according to the ranking system proposed in Smart-Plexer 1.0.^12^ It is also interesting to see that PM7.14 is ranked at the top by both strategies.

Figure 5 illustrates the detailed ACA classification performance for selected assays through confusion matrices and corresponding t-distributed Stochastic Neighbor Embedding (t-SNE) mappings.^23^ The classification accuracies and MSS of the t-SNE mapped features are given in the plot titles. Clusters were mapped by t-SNE, and the colors were presented with target labels. t-SNE technique visualizes the feature distributions using manifold learning,^23^ with smaller cluster contours and larger intertarget cluster distances indicating easier separability by nonlinear classifiers. Here, MSS evaluates clustering quality with higher MSS reflecting better-defined clusters, indicating easier ACA classification with the given assay.

**Figure 5:**
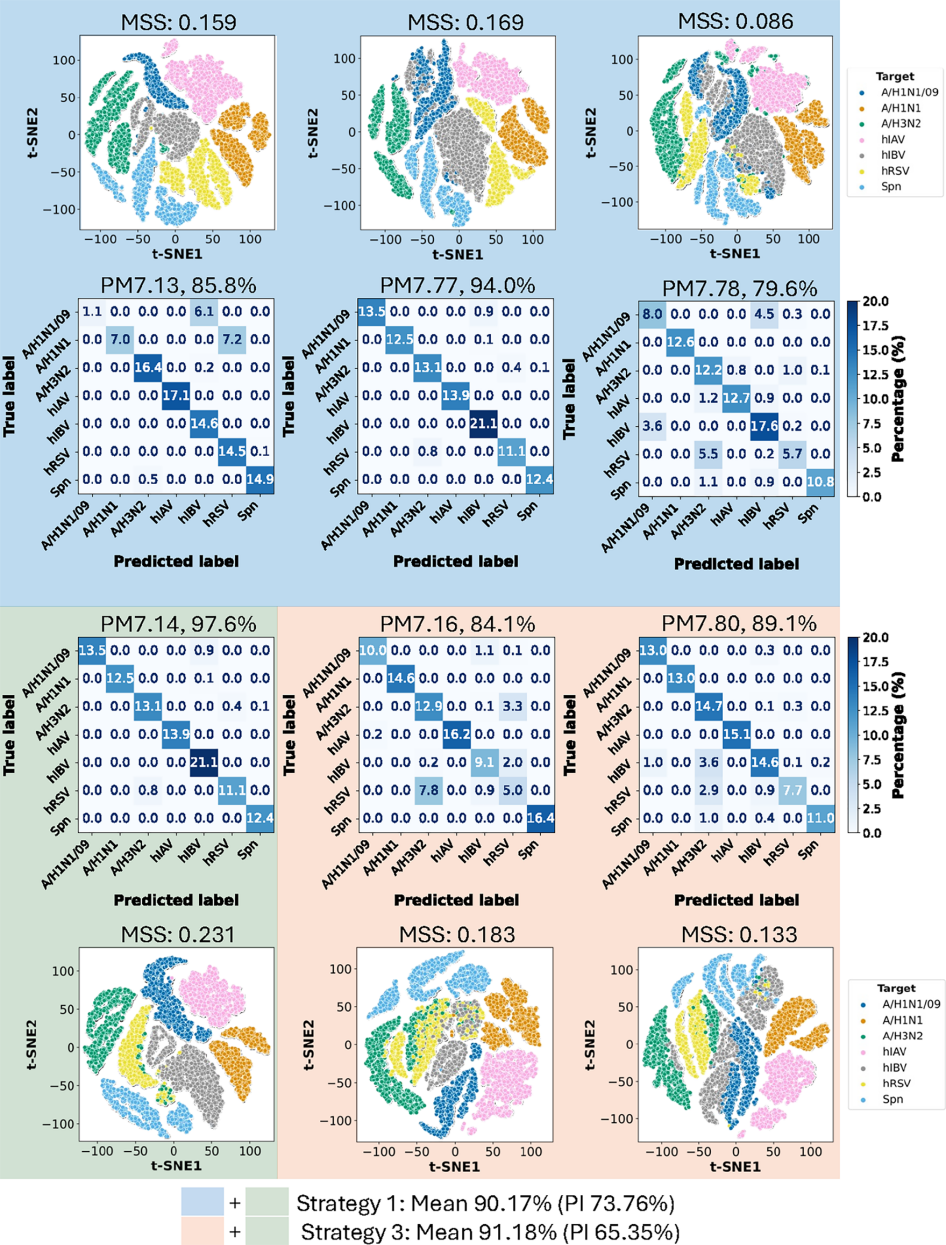
ACA performance of assays selected by S1 and S3 on the SP2-7-plex-RTI data set. S1-picked, S3-picked, and commonly picked assays are marked with different colors. For each assay, the confusion matrix with the ACA accuracy and the t-SNE mapping with the Mean Silhouette Scores of target clusters are presented in a stacked way.

As depicted in Figure 5, PM7.14 demonstrates the highest target identification accuracy, with most samples falling into the diagonal of the confusion matrix. Also, its t-SNE mapping clearly separates target clusters, reflecting distinct boundaries and large intercluster distances. PM7.78 shows the opposite performance with ACA accuracy less than 80% and MSS close to zero (indicating that clusters are indifferent). In the t-SNE mapping, broken clusters of A/H1N1/09 and hRSV can be spotted. All the other primer mixes have accuracies between 84.1 and 94%, and on average, the S3 picked assays are showing better and more robust performance, with over 1% higher mean ACA accuracy, 8.41% smaller PI, and larger mean MSS (0.161 for S1 vs 0.182 for S2).

### Improvement of Smart-Plexer 2.0

This research aims to enhance Smart-Plexer 1.0 by incorporating additional kinetic features alongside the “*c*” parameter and introducing a clustering-based distance calculation, thereby improving its robustness under varying experimental conditions such as concentration fluctuations and PCR inhibitors. Our results indicate that while the S1 strategy can still identify top-performing primer mixes, the expanded feature set and clustering-based distance measurement of S3 provide a more stable and reliable prediction of optimal combinations. Incorporating comprehensive kinetic and target-distribution information significantly increases the likelihood of selecting high-performance assays while avoiding less promising combinations. This improvement is evident in the reduced interval between the maximum and minimum classification accuracy (18% accuracy difference for S1 and 13.5% for S3), demonstrating a more consistent performance.

Practically, multiplex assay design faces challenges due to limited empirical testing capacity resulting from cost and time constraints. Smart-Plexer 2.0 addresses these challenges by efficiently and accurately identifying promising primer combinations in silico, significantly streamlining assay development. This advancement offers a tangible benefit in diagnostics, where a rapid and precise assay design is crucial.

Comparing with existing curve-derived sigmoidal parameters, the selected features show significant advantages: (1) they are calculated on top of the 5-parameter model, presenting complex nonlinear relations inside AC, also avoiding notches and artifacts which usually appear in high-level features; (2) they incorporate both slope (first-derivative) and second-derivative changing trends, taking asymmetry between exponential and plateauing phases into consideration; (3) they are explainable from the perspective of molecular biology, enabling further analysis and optimization of chemistry.

### Considerations in Clinical Applications

The proposed strategy demonstrates a moderate increase in ACA accuracy, as the assays are already well-designed with a high benchmark accuracy, leaving little room for further improvement in the numbers. However, statistical tests show clear significance, making the comparison results reliable. In clinical practice, ACA in dPCR uses a “panel ensemble” strategy, where predictions from all wells within a panel are combined for final sample classification, improving accuracy by 4–14% based on prior validations.^10^ Thus, the best assays in Figure 5 may achieve nearly 100% sample-level accuracy with dPCR in real clinical applications. However, applying the same models to qPCR may reduce performance due to platform-specific data distribution differences. Addressing this domain shift, potentially using our published T-CDAN method,^10^ will be the focus of future work.

Explainable models, such as Random Forest, can effectively indicate feature importance when training and testing data distributions align. However, since our singleplex PCR data differ significantly in distribution from multiplex data, showing correlation but not numerical equivalence, such models can become biased, causing inconsistent and suboptimal feature selection. To address this domain discrepancy, we instead evaluated features directly using clustering and statistical tests, ensuring robust feature selection without domain-shift bias.

Theoretically, there may be differences between data from single-molecule digital PCR and conventional bulk-reaction qPCR (multiple molecules per reaction). Although most results presented were obtained using digital PCR, we anticipate comparable performance of features with qPCR (retraining of classification models may be required), given that these features consistently demonstrated concentration robustness in the Data set Ex-Conc-Eff, which included external qPCR data. Further efforts will focus on evaluating this hypothesis.

Simulating multiplexes with singleplex reactions can introduce artifacts and reduce efficiency due to primer interactions. To address this, NTC tests were performed to eliminate primer dimers and nonspecific products. Previous work showed that although feature values may shift between singleplex and multiplex, carefully selected features preserve intertarget distance information through strong linear correlations.^12^ This is also validated in Figure 3c for all chosen features, ensuring that target distances remain consistent and that selected assays remain effective.

### Limitations and Future Directions

First, the accuracy of Smart-Plexer’s predictions heavily depends on the quality of input data from singleplex assays. Inaccuracies may yield suboptimal multiplex designs. Second, although designed for multiplex PCR complexity, Smart-Plexer 2.0 still encounters challenges with highly complex interactions, such as primer competition or nonspecific amplification, which are challenging to predict in silico. Further improvements, including detailed modeling of primer interactions and in-silico simulations incorporating PCR inhibitors, are required to address these issues.^16,24^

Additionally, this study primarily focuses on assay design, without extensive investigation into the detection limits for ultralow concentration targets or the performance with high-GC content templates. For ultralow target concentrations, sensitivity is fundamentally constrained by amplification efficiency and stochastic effects at low copy numbers, potentially requiring further optimization of primer/probe design or preamplification strategies. For high-GC samples, inefficient denaturation can adversely affect performance; thus, future work will systematically evaluate these challenges and explore protocol modifications to improve the assay robustness.

Furthermore, the computational demands of Smart-Plexer 2.0, particularly with the incorporation of new features and advanced distance measurements, can be intensive, requiring substantial processing time and resources. Tables S4 and S5 summarize the running time for each step of the Smart-Plexer and ACA algorithms. On average, Smart-Plexer requires 4.6 ms per reaction under Strategies 1 and 2, and 10.8 ms under Strategy 3 due to the more computationally intensive clustering distance calculations. For ACA, generating a target prediction for each selected assay takes only 5.5 ms. Although it is practical for real-time clinical applications under the provided computing environment, the intensive calculation required can limit its scalability and practicality in resource-limited laboratory settings. To mitigate this, we are working on transforming Smart-Plexer 2.0 into a cloud-based software solution that optimizes memory usage, allowing users to handle all computational tasks through an Internet connection, thereby broadening its accessibility and ease of use in diverse laboratory environments.

## Conclusions

The study presented significant advancements in developing and optimizing multiplex PCR assays, particularly through the enhancement of the Smart-Plexer framework. By introducing new features extracted from amplification curves, such as those related to reaction efficiency and curve asymmetry, the study addressed the limitations of Smart-Plexer 1.0, which relied merely on the “*c*” parameter. New features have demonstrated robustness across various concentrations and reaction efficiencies, which are crucial for reliable assay development under multiplex conditions. We also proposed the clustering-based distance measurement for ADS and MDS calculations, aiming for a more inclusive consideration of intertarget distances.

The improved Smart-Plexer 2.0 framework, with its extended feature set and clustering-based distance measurement, outperforms the original version by providing a more stable and reliable prediction of optimal primer combinations. This enhanced approach improves the consistency of assay selection and increases the likelihood of identifying high-performing assays while minimizing the risk of selecting suboptimal ones. The successful application of this methodology in the design of a new 7-plex assay for respiratory tract infections further validates its practical utility.

In conclusion, Smart-Plexer 2.0 offers a powerful tool for the rapid and accurate design of multiplex assays, addressing the challenges posed by target concentration fluctuations and PCR inhibitors. This advancement is particularly valuable in diagnostic settings, where time and accuracy are critical, and it represents a significant step forward in the field of multiplex PCR assay development.

## Supplementary Material



## Notes

Data set description; materials and methods; development environment and running time; feature selection; and ranking accuracy results (PDF)
