# Supplementary material for: Smart-Plexer 2.0: Leveraging New Features of Amplification Curves to Enhance the Selection of Multiplex PCR Assays in Multi-Target Identification
Source: Anal Chem. 2025 Jul 2;97(27):14311–20. doi: 10.1021/acs.analchem.5c01181 (PMC12268814; doi:10.1021/acs.analchem.5c01181)
Supplement: Supplementary file 1 [file ac5c01181_si_001.pdf]

Supporting Information

Smart-Plexer 2.0: Leveraging New Features of Amplification Curves to Enhance the Selection of Multiplex PCR Assays in Multi-Target Identification

Ke Xu[a,b,†], Luca Miglietta[a,†], Piyanate Kesakomol[a], Alison Holmes[a], Pantelis Georgiou[b], Nicolas Moser[b], Jesus Rodriguez-Manzano[a,*]

[a] Department of Infectious Disease, Faculty of Medicine, Imperial College London, London, UK
[b] Department of Electrical and Electronic Engineering, Faculty of Engineering, Imperial College London, London, UK
[†] These authors contributed equally to this work.
*Corresponding author: j.rodriguez-manzano@imperial.ac.uk

Table of Contents

Section S1. Dataset Description S-2
Section S2. Materials and Methods S-3
Table S1 Primer Table S-4
Table S2 Definition and Description of Proposed New Features S-6
Table S3 Key Packages of the Development Environment S-7
Table S4 Running Time for ACA Algorithms (41128 Reactions) S-7
Table S5 Running Time for Smart-Plexer Algorithms (34650 Reactions) S-7
Figure S1. Feature distributions across concentrations on SP1-7-plex-RTI dataset S-8
Figure S2. Feature distribution consistency among concentrations under different reaction efficiencies tested on the Ex-Conc-Eff dataset S-9
Figure S3. Ranking-accuracy mapping of SP1-3-plex-RTI dataset S-10
Reference S-11

Section S1. Dataset Description

To ensure robustness, both retrospective and newly generated datasets with cross-platform, cross-experiment, and cross-concentration characteristics were employed.

(I) The 7-plex Respiratory Tract Infection (RTI) dataset (SP1-7-plex-RTI): Acquired from our previous Smart-Plexer 1.0 research, it contains reactions of Human adenovirus (HAdV), Human coronavirus OC43 (HCoV-OC43), Human coronavirus HKU1 (HCoV-HKU1), Human coronavirus 229E (HCoV-229E), Human coronavirus NL63 (HCoV-NL63), Middle East respiratory syndrome-related coronavirus (MERS-CoV), and Severe acute respiratory syndrome coronavirus 2 (SARS-CoV-2) in a single fluorescent channel using real-time digital PCR (qdPCR), across up to 5 concentrations ($10^3, 10^4, 10^5, 10^6, 10^7$ copies/µL), with 36,960 reactions in total. It was used for developing and selecting optimal features from the amplification curve (AC).

(II) External concentration-efficiency dataset (Ex-Conc-Eff): In this external open dataset, real-time PCR (qPCR) reactions were conducted with the MT-ND1 gene over a wide range of input DNA ($3.14 \times 10^1 - 3.14 \times 10^7$ molecules) with amplification master mix quantities ranging from 60% to 100% of the optimal amount. This dataset, which contains 420 amplification curves, produced different reaction kinetics under a variety of DNA concentrations, mimicking the amplification efficiency reduction that happens during multiplex reactions in which primer sets and probes of several targets are mixed. We leverage this external dataset to validate the robustness and consistency of the proposed new AC kinetic features across concentrations in extreme reaction conditions.

(III) The 3-plex RTI dataset (SP1-3-plex-RTI): The three targets were from our previous Smart-Plexer research, including Adenovirus (HAdV), Human coronavirus HKU1 (HCoV-HKU1) and the Middle East respiratory syndrome-related coronavirus (MERS-CoV). This retrospective dataset, with 62,370 reactions, was involved in this study because all 27 possible combinations of multiplex assays of the three targets were exhaustively tested empirically in multiplex conditions, making it possible to conduct an overall comparison among different assay selection strategies. This previous 3-plex RTI dataset was used for statistical evaluation of the previous and the new Smart-Plexer strategies over different AC features and distance calculations.

(IV) New 7-plex RTI dataset (SP2-7-plex-RTI): To further prove the effectiveness of the improved Smart-Plexer framework, we applied both the original and proposed new Smart-Plexer strategies on a newly designed multiplex panel of 7 respiratory infectious pathogens: Influenza A Virus H1N1 2009 or Swine Flu (A/H1N1/09), Influenza A Virus H1N1 (A/H1N1), Influenza A Virus H3N2 (A/H3H2), Influenza A Virus (hIAV), Influenza B Virus (hIBV), Respiratory Syncytial Virus (hRSV), and Streptococcus pneumoniae (Spn). The candidate primer sets and probes (1-3 per target) were designed for each target, resulting in 128 possible multiplex combinations. Experiments were first conducted in singleplex with real-time digital PCR (qdPCR) for each assay to get the simulated multiplex AC required for Smart-Plexer, generating 34,650 ACs in total. After applying the old and new Smart-Plexer strategies, 6 optimal multiplex combinations were selected, for which single-well multiplex reactions were performed in qdPCR with a range of different target concentrations ($10^4, 10^5, 10^6, 10^7$ copies/µL). A total of 18 tests with separate qdPCR chips were conducted for the empirical multiplex, resulting in 665,280 amplification curves. Details of experiment conditions and assays can be found in **Section S2**.

Our previously proposed Adaptive Mapping Filtering, which is an unsupervised pre-processing algorithm for eliminating NTC, flat curves, non-specific, and low-efficient reactions from qdPCR amplification curves, was applied to all the qdPCR datasets mentioned above.

Section S2. Materials and Methods

Double-stranded synthetic DNA was used in this study to develop and assess the performance of all singleplex assays. In particular, we used the entire coding sequence of the following strains: Influenza A Virus H1N1 2009 or Swine Flu (A/H1N1/09), Influenza A Virus H1N1 (A/H1N1), Influenza A Virus H3N2 (A/H3N2), Influenza A Virus (hIAV), Influenza B Virus (hIBV), Respiratory Syncytial Virus (hRSV), Streptococcus pneumoniae (Spn). The following NCBI accession numbers were used as references for the gBlocks synthesis: CY181417, MN055282, NC_007366, NC_002016, NC_002204, NC_001803, NC_003098, respectively. Based on the comprehensive analyses and alignments of each type using the MUSCLE algorithm[1], primers were specifically designed to amplify all sequence variations within each gene belonging to their specific target (inclusivity) and to exclude closely related but not inclusive sequences (exclusivity). Design and in-silico analysis were conducted using Geneious Prime 2022.0.1[2]. Oligonucleotides and synthetic constructs (length ranging from 747 to 1,775 bp) were purchased from Integrated DNA Technologies Ltd. (IDT) and resuspended in Tris−EDTA buffer to 10 ng/μl stock solutions (stored at −80°C until further use). The concentrations of all DNA stock solutions were determined using a Qubit 3.0 fluorimeter (Life Technologies).

For real-time amplification experiments, we used the BioMark HD (Fluidigm). The master mix used was the PrimeTime Gene Expression Master Mix from Integrated DNA Technologies (IDT, catalog no. 1055772) supplemented with ROX passive reference dye and pre-mixed following the manufacturer's guidelines. The qdPCR was performed with Fluidigm qdPCR 37k integrated fluidic circuits (IFC) (catalog no. SKU100-6152) and was supplemented with Fluidigm 20X GE loading buffer (PN 85000746). The priming and loading steps of the IFC were followed as the supplier's protocol (Fluidigm document number: 100-6896 Rev 03). Each amplification mix for the qdPCR experiment contained 3 μl 2X IDT PrimeTime Gene Expression Master Mix (with passive ROX), 0.6 μl 20X GE, 0.6 μl 10X Primer mixture, 1.8 μl DNA templates from synthetic DNA, pre-amplified cDNA, or controls, and to bring the final volume to 6 μl. A total of 4.5 μl of reaction mix was transferred to each inlet (or panel) of a Fluidigm 37k IFC for the thermal cycling step. Thermal-cycle conditions consisted of a hot start step for 3 min at 95 °C, followed by 45 cycles at 95 °C for 15 seconds and 60 °C for 45 seconds. Real-time data of the amplification events were exported as a text file for each bulk by Fluidigm Digital PCR Analysis software (version 4.1.2)

Table S1 Primer Table[3]

primer_id	primer sequence	strain
9H101	TACCCAAARCTCAGCAAATCCT	A/H1N1/09
9H103	AGACTTTGTTGGTCAGCACTAGT	A/H1N1/09
AH101	AATTGGGTAAYTGCAGCGTTGC	A/H1N1
AH103	GTKTTTCTACAATGTAGGACCATG	A/H1N1
AH104	CCCTGGGTAACATGTTCCATTCTC	A/H1N1
AH301	AGCAACTGTTACCCTTATGATGT	A/H3N2
AH302	TATGATGTGCCGGATTATGCCT	A/H3N2
AH304	CAGTCCAATTGAAGCTTTCATTGT	A/H3N2
AH305	TGAGTGACTCCAGTCCAATTGA	A/H3N2
FLA01	TGCAGTCCTCGCTCACTGGGCACG	hIAV
FLA02	TGCAGTCCTCGCTCACTGGGCACG	hIAV
FLA16	ACCAATCCTGTCACCTCTGACT	hIAV
FLA17	GGCATTTTGGACAAAGCGTCT	hIAV
FLB01	GATCGAATCTGCACTGGRATAAC	hIBV
FLB03	TGCTACTCAAGGGGARGTCAA	hIBV
FLB05	GTGGGTGTTGTTGTCAGTGGTAT	hIBV
FLB07	CCYCTGGTTTCTGTTCCTTTGA	hIBV
FLB08	GGCTACRTCCAGATCTGTGCA	hIBV
RSV01	GCAAATATGGAAACATACGTGAACA	hRSV
RSV03	GCACCCATATTGTWAGTGATGCA	hRSV
RSV04	AAAGACGATGAYCCTGCATC	hRSV
RSV06	AGCACTGCACTTCTYGAGTT	hRSV
SPN01	ACGCAATCTAGCAGATGAAGCA	Spn
SPN03	CTCGTGCGTTTTAATTCCAGCT	Spn

Target	gBlocks	Assay_ID	Forward_ID	Probe_ID	Reverse_ID	Comments
9H1	A2009_HA_x_CY181417	9H1_HA_02	9H101	9H104	9H103	
9H1	A2009_HA_x_CY181417	9H1_HA_03	9H101	9H105	9H103	
AH1	AH1N1_HA_x_MN055282	AH1_HA_03	AH101	AH105	AH103	
AH1	AH1N1_HA_x_MN055282	AH1_HA_04	AH101	AH105	AH104	
AH1	AH1N1_HA_x_MN055282	AH1_HA_05	AH101	AH106	AH103	
AH1	AH1N1_HA_x_MN055282	AH1_HA_06	AH101	AH106	AH104	
AH3	AH3N2_HA_x_NC_007366	AH3_HA_03	AH301	AH307	AH304	
AH3	AH3N2_HA_x_NC_007366	AH3_HA_04	AH302	AH307	AH305	
RSV	ReSyV_M1_x_NC_001803	RSV_M1_03	RSV01	RSV07	RSV03	
RSV	ReSyV_M1_x_NC_001803	RSV_M1_04	RSV04	RSV08	RSV06	
SPN	Strpn_lytA_x_NC_003098	SPN_LYT_02	SPN01	SPN04	SPN03	
FLB		FLB_HA_05	FLB01	FLB09	FLB05	

FLB		FLB_HA_06	FLB03	FLB10	FLB05	
FLB		FLB_HA_07	FLB01	FLB11	FLB07	
**FLB**		**FLB_HA_08**	**FLB03**	**FLB11**	**FLB08**	**NOT USED**
FLA	Flu-A_M12_x_NC_002016	FLA_M_01	FLA16	FLA01	FLA17	
FLA	Flu-A_M12_x_NC_002016	FLA_M_02	FLA16	FLA02	FLA17	

	**9H1**	**AH1**	**AH3**	**FLA**	**FLB**	**RSV**	**SPN**
**PM7.078**	9H1_HA_03	AH1_HA_03	AH3_HA_04	FLA_M_02	FLB_HA_05	RSV_M1_04	SPN_LYT_02
**PM7.013**	9H1_HA_02	AH1_HA_03	AH3_HA_04	FLA_M_02	FLB_HA_05	RSV_M1_03	SPN_LYT_02
**PM7.014**	9H1_HA_02	AH1_HA_03	AH3_HA_04	FLA_M_02	FLB_HA_05	RSV_M1_04	SPN_LYT_02
**PM7.077**	9H1_HA_03	AH1_HA_03	AH3_HA_04	FLA_M_02	FLB_HA_05	RSV_M1_03	SPN_LYT_02
**PM7.016**	9H1_HA_02	AH1_HA_03	AH3_HA_04	FLA_M_02	FLB_HA_07	RSV_M1_04	SPN_LYT_02
**PM7.080**	9H1_HA_03	AH1_HA_03	AH3_HA_04	FLA_M_02	FLB_HA_07	RSV_M1_04	SPN_LYT_02

Table S2 Definition and Description of Proposed New Features

Name	Definition	Description
Threshold Distance	$x_e - x_s$	**Mathematical**: Distance between the two Threshold-crossing locations in the first derivative. **PCR curve**: Difference between the cycle number (or time) at the beginning of the exponential phase to the beginning of the plateau phase.
First-half Distance	$x_{ms} - x_s$	**Mathematical**: Distance between the first Threshold-crossing location and the maximum-slope location in the first derivative. **PCR curve**: Difference between the cycle number (or time) at the beginning of the exponential phase to the cycle number (or time) with the fastest increase of fluorescence value.
Second-half Distance	$x_e - x_{ms}$	**Mathematical**: Distance between the second Threshold-crossing location and the maximum-slope location in the first derivative. **PCR curve**: Difference between the cycle number (or time) with the fastest increase of fluorescence value to the beginning of the plateau phase.
Distance Asymmetrical Index	$\frac{x_e - x_{ms}}{x_{ms} - x_s}$	**Mathematical**: Ratio of Second-half Distance to First-half Distance, indicating how asymmetrical the peak is in the two phases of the derivative of the fitted curve. **PCR curve**: Indication of curve asymmetry between the beginning and ending phase of the reaction.
Peak-shifting Distance	$x_{p2} - x_{p1}$	Distance between the positive and negative peak locations in the second derivative
A_1	$\sum_{x=x_s}^{x_{ms}} f'(x)$	Integration of the first derivative from x_s to x_{ms}, indicating the area under the first half of the peak
A_2	$\sum_{x=x_{ms}}^{x_e} f'(x)$	Integration of the first derivative from x_{ms} to x_e, indicating the area under the second half of the peak
Area Asymmetrical Index	$\frac{A_2}{A_1}$	**Mathematical**: Ratio of A_2 to A_1 , indicating how asymmetrical the area-under-peak is **PCR curve**: Indication of curve asymmetry between the beginning and ending phase of the reaction.
Maximum Slope	$\frac{df}{dx}\vert_{x_{ms}}$	**Mathematical**: Height of the largest peak in the first derivative. **PCR curve**: The largest fluorescence value increased between two continuous cycles.
Positive Second-derivative Peak Height	$\frac{d^2f}{dx^2}\vert_{x_{p1}}$	**Mathematical**: Height of the positive peak on the second derivative, **PCR curve**: The reaction speed at the beginning phase of PCR
Negative Second-derivative Peak Height	$-\frac{d^2f}{dx^2}\vert_{x_{p2}}$	**Mathematical**: Absolute height of the negative peak on the second derivative. **PCR curve**: The reaction speed at the beginning of the plateau phase.
Peak Asymmetrical Index	$-\frac{\frac{d^2f}{dx^2}\vert_{x_{p1}}}{\frac{d^2f}{dx^2}\vert_{x_{p2}}}$	**Mathematical**: Ratio of the positive peak height to the negative peak height in the second derivative. **PCR curve**: Indication of curve asymmetry between the beginning and ending phase of the reaction.

Table S3 Key Packages of the Development Environment

Name	Version
python	3.9.17
numpy	1.22.4
pandas	1.5.3
matplotlib	3.7.1
seaborn	0.12.2
scipy	1.10.1
scikit-learn	1.2.2

Table S4 Running Time for Smart-Plexer Algorithms (34650 Reactions)

Name	Time Period (s)
Pre-Processing & Sigmoidal Fitting	148.6
Adaptive Mapping Filtering	4.3
Feature Extraction	4.2
Distance Calculation (S1)	0.8
Distance Calculation (S2)	1
Distance Calculation (S3)	217.3
Ranking	0.1

Table S5 Running Time for ACA Algorithms (41128 Reactions)

Name	Time Period (s)
Pre-Processing & Sigmoidal Fitting	194.2
Adaptive Mapping Filtering	4.8
Feature Extraction	4
Feature Normalization	22.1
Classification	1.8

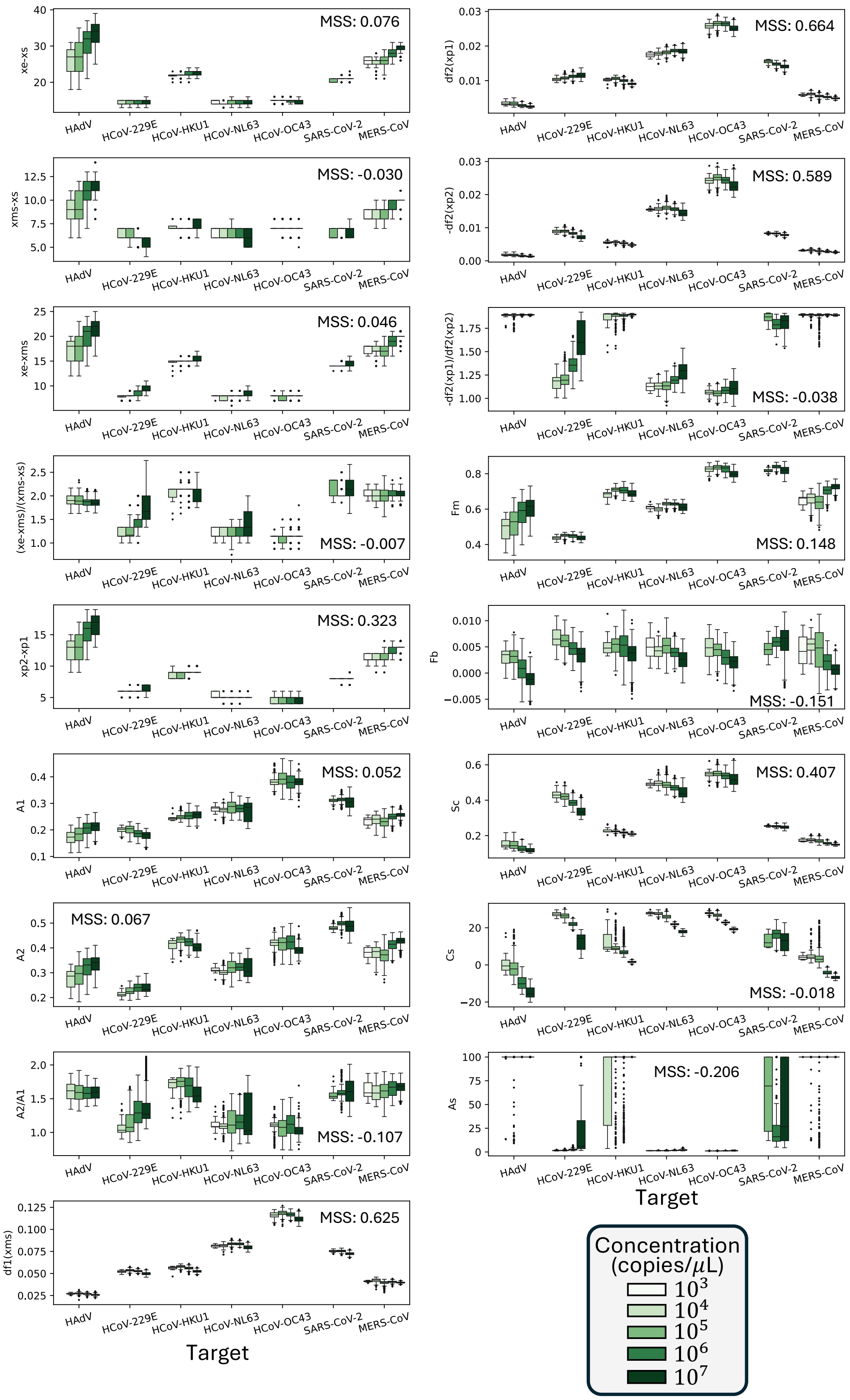

Figure S1. The feature distributions across concentrations on SP1-7-plex-RTI dataset. Box plots of various target types and concentrations are shown. The X-axis shows different pathogen DNA targets, and distributions of different target concentrations are depicted in shades of colors and grouped in subplots. Mean Silhouette Scores among target clusters are also presented in the upper-right corner, for each feature.

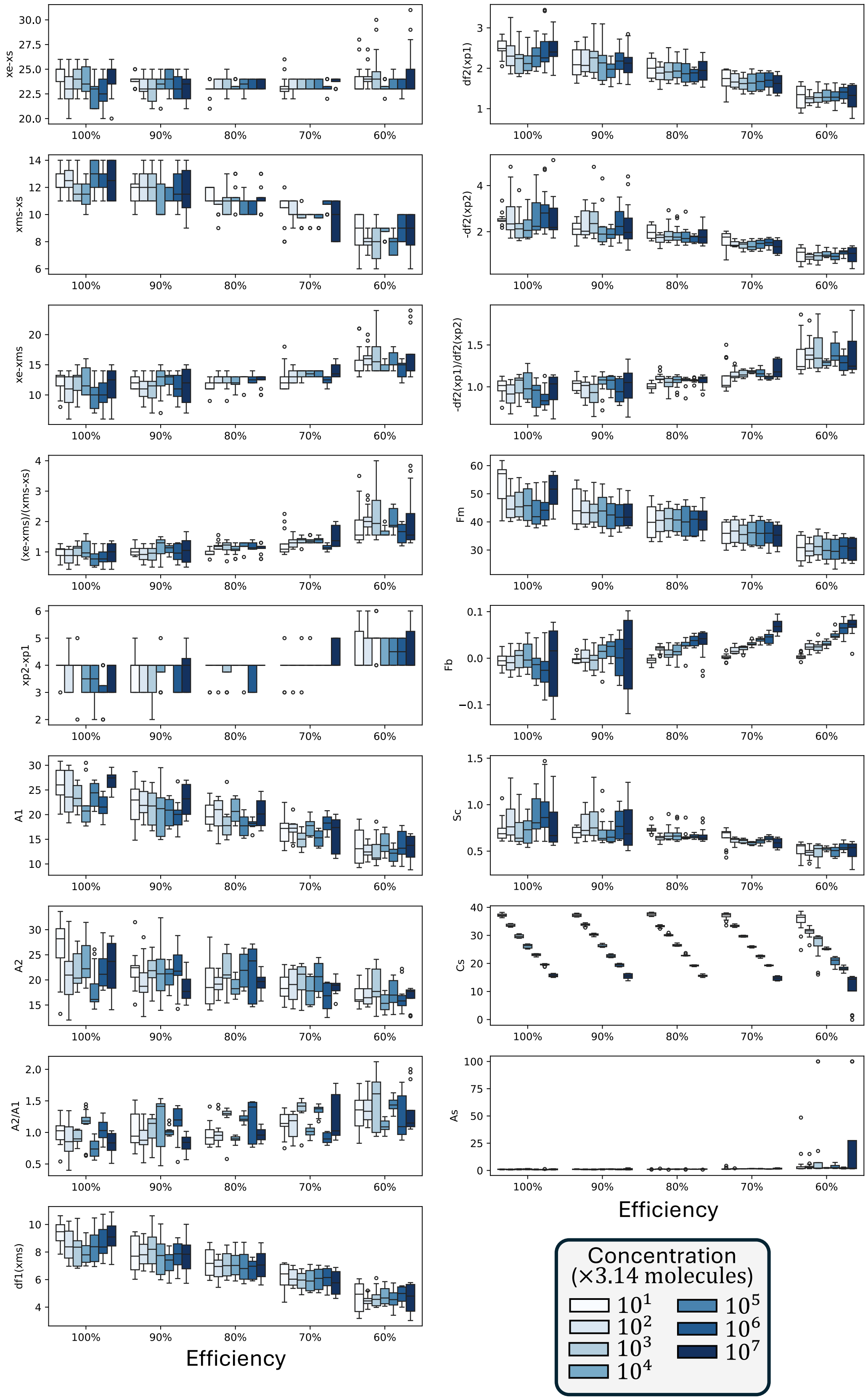

Figure S2. Feature distribution consistency among concentrations under different reaction efficiencies tested on the Ex-Conc-Eff dataset. Box plots of various target concentrations and reaction efficiencies are shown. The X-axis shows different reaction efficiencies, and distributions of different template concentrations are depicted in shades of colors and grouped in subplots.

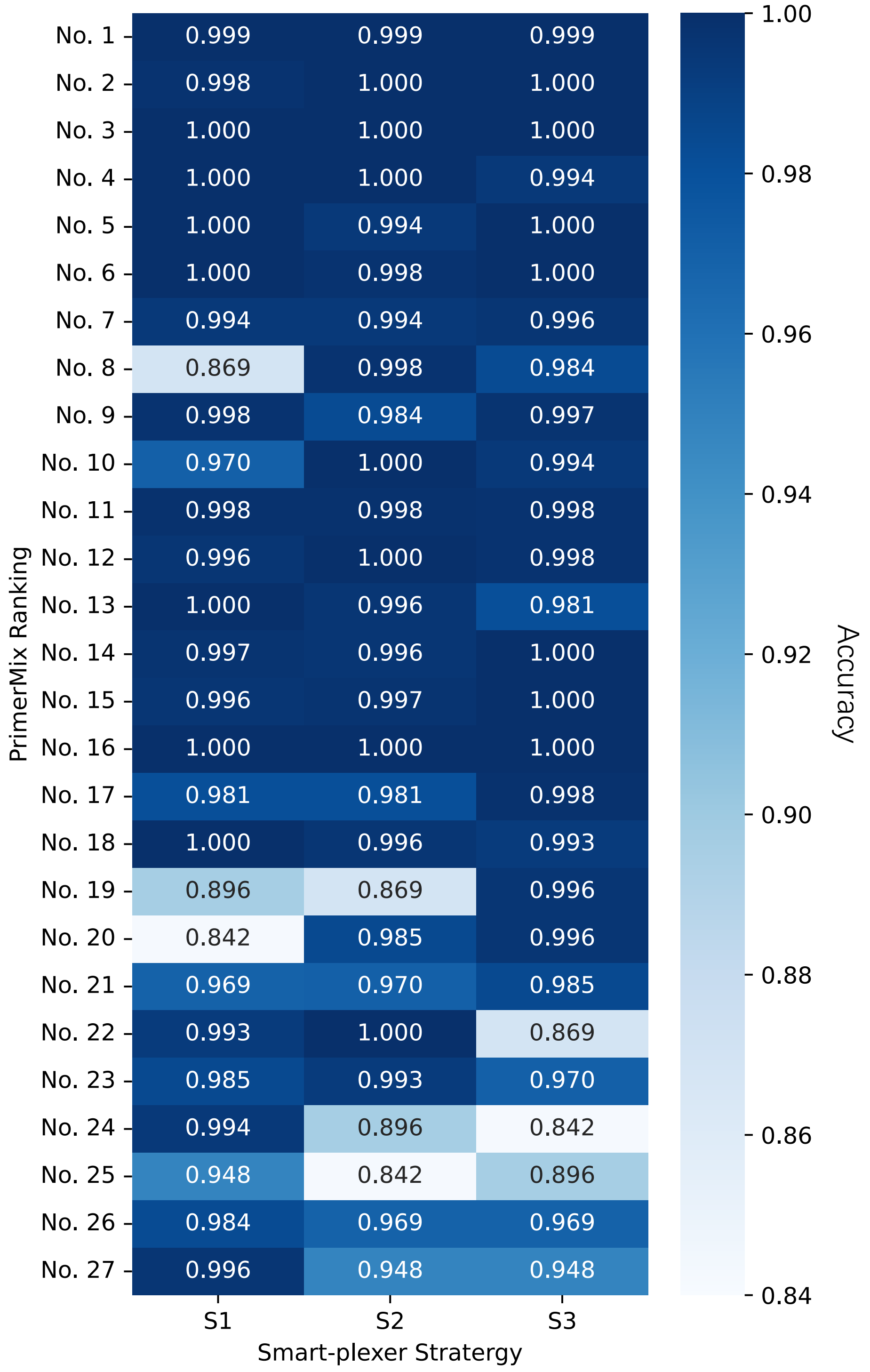

Figure S3. Ranking-accuracy mapping of SP1-3-plex-RTI dataset, for three strategies. From top to bottom, each column shows the real-world ACA accuracies of the multiplexes ranked from the first to the last, in both numerical values and colors. Ideally, the higher the rank, the better performance and accuracy it will present. S2 and S2 follow this assumption, but S1 shows a complex pattern with highly ranked assays providing unexpectedly worse ACA performance, e.g., No. 8.

Reference

1. Edgar, R. C. MUSCLE: multiple sequence alignment with high accuracy and high throughput. *Nucleic Acids Research* **32**, 1792–1797 (2004).
2. Kearse, M. *et al.* Geneious Basic: An integrated and extendable desktop software platform for the organization and analysis of sequence data. *Bioinformatics* **28**, 1647–1649 (2012).
3. Miglietta, L. The art of PCR assay development: data-driven multiplexing. (Imperial College London, 2023).
